# In Vitro Evaluation of Third Generation PAMAM Dendrimer Conjugates

**DOI:** 10.3390/molecules22101661

**Published:** 2017-10-04

**Authors:** Mohammad Najlah, Sally Freeman, Mouhamad Khoder, David Attwood, Antony D’Emanuele

**Affiliations:** 1 Faculty of Medical Science, Anglia Ruskin University, Bishops Hall Lane, Chelmsford CM1 1SQ, UK; 2 Division of Pharmacy and Optometry, School of Health Sciences, Manchester Academic Health Sciences Centre, University of Manchester, Manchester M13 9PL, UK; sally.freeman@manchester.ac.uk (S.F.); David.Attwood@manchester.ac.uk (D.A.); 3 School of Life Sciences, Pharmacy and Chemistry, Kingston University London, Kingston upon Thames KT1 2EE, UK; m.khoder@kingston.ac.uk; 4 Leicester School of Pharmacy, De Montfort University, Leicester LE1 9BH, UK; antony.demanuele@dmu.ac.uk

**Keywords:** PAMAM dendrimers, transepithelial transport, Caco-2 cells, dendrimer prodrugs, oral drug delivery

## Abstract

The present study compares the use of high generation G3 and low generation G0 Polyamidoamine (PAMAM) dendrimers as drug carriers of naproxen (NAP), a poorly water soluble drug. Naproxen was conjugated to G3 in different ratios and to G0 in a 1:1 ratio via a diethylene glycol linker. A lauroyl chain (L), a lipophilic permeability enhancer, was attached to G3 and G0 prodrugs. The G3 and G0 conjugates were more hydrophilic than naproxen as evaluated by the measurement of partitioning between 1-octanol and a phosphate buffer at pH 7.4 and pH 1.2. The unmodified surface PAMAM-NAP conjugates showed significant solubility enhancements of NAP at pH 1.2; however, with the number of NAP conjugated to G3, this was limited to 10 molecules. The lactate dehydrogenase (LDH) assay indicated that the G3 dendrimer conjugates had a concentration dependent toxicity towards Caco-2 cells. Attaching naproxen to the surface of the dendrimer increased the IC_50_ of the resulting prodrugs towards Caco-2 cells. The lauroyl G3 conjugates showed the highest toxicity amongst the PAMAM dendrimer conjugates investigated and were significantly more toxic than the lauroyl-G0-naproxen conjugates. The permeability of naproxen across monolayers of Caco-2 cells was significantly increased by its conjugation to either G3 or G0 PAMAM dendrimers. Lauroyl-G0 conjugates displayed considerably lower cytotoxicity than G3 conjugates and may be preferable for use as a drug carrier for low soluble drugs such as naproxen.

## 1. Introduction

Dendrimers represent highly branched macromolecules that have a well-defined structure with precisely controlled size and shape as well as terminal group functionality [1]. The potential use of dendrimers in several pharmaceutical applications including controlled drug delivery has been extensively investigated [2,3,4]. The high degree of branching allows drug encapsulation and the formation of dendrimer-drug conjugates [5], in addition, the ability to modify terminal functional groups allows for the surface engineering of dendrimers for different applications such as the enhancement of drug solubility [6,7,8] and permeability [9].

In dendrimer-drug conjugates, the drug is combined through a covalent bond either directly or via a linker/spacer to the dendrimer. Our previous work showed that the direct conjugation of a drug to PAMAM dendrimer (via an amide bond) resulted in a stable conjugate that would not be suitable for use as a drug delivery system [10]. In contrast, using diethylene glycol as a linker/spacer between the drug and dendrimer resulted in ester prodrugs that showed high chemical stability at pH 1.2, 7.4 and 8.5 (37 °C), but that readily released the drug in human plasma (in vitro). Such conjugates have potential as carriers for low solubility drugs, such as naproxen. More recently, surface modified G0-naproxen conjugates were found to increase significantly the permeability of naproxen across epithelial cells [11].

**Figure 1:**
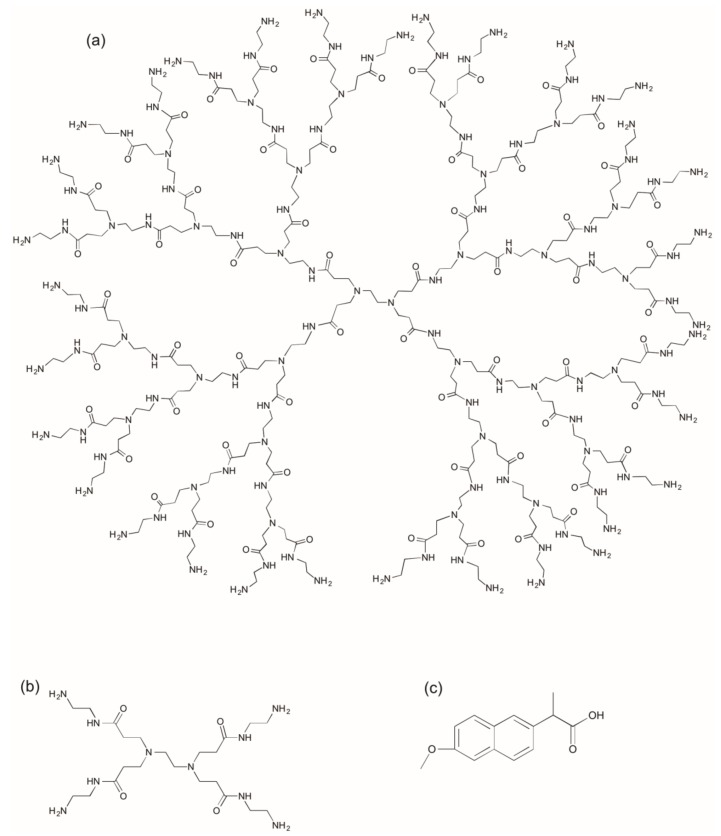
Structure of (**a**) G3 PAMAM dendrimer with an ethylenediamine core, (**b**) G0 PAMAM dendrimer with an ethylenediamine core and (**c**) Naproxen.

In the present study, naproxen (Figure 1) is conjugated to both G0 and G3 PAMAM dendrimers, via a diethylene glycol linker. The biological properties (in vitro) of the conjugates are compared including their cytotoxicity (LDH assay) and transport across Caco-2 monolayers. The influence of attaching a surface modifier (lauroyl chain) on transport properties is also evaluated and the respective merits of using G0 and G3 as oral drug carriers are considered.

## 2. Results and Discussion

### 2.1. Synthesis and Characterization of G3 PAMAM Dendrimer-Naproxen Conjugates

**Figure 2:**
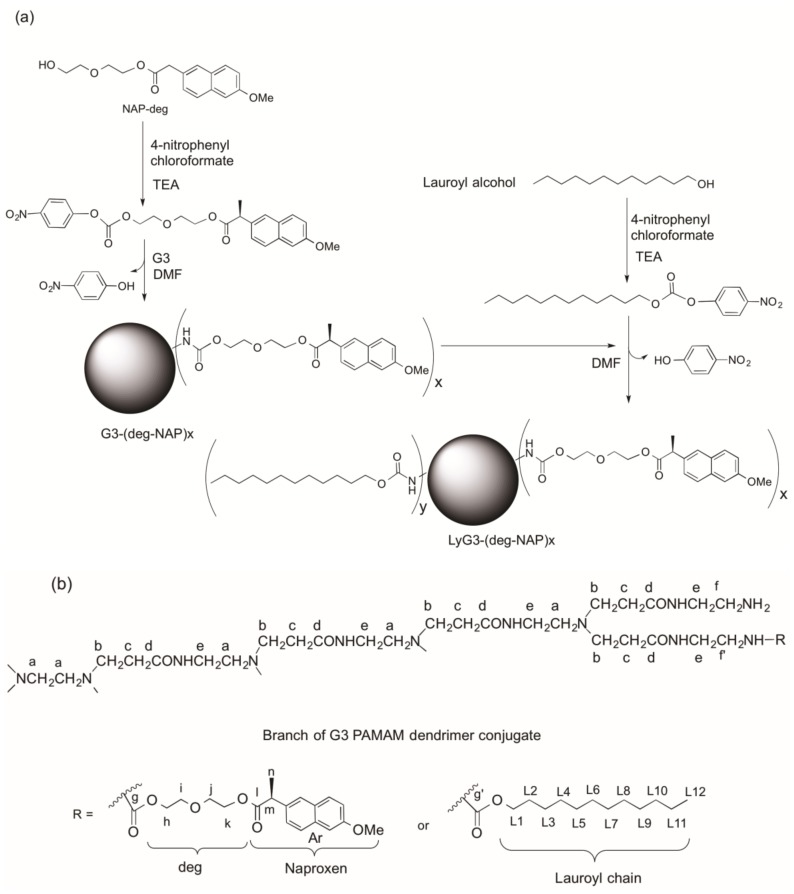
(**a**) Illustrative scheme for the synthesis of G3 PAMAM conjugates and (**b**) G3 PAMAM dendrimer-naproxen conjugates (letters are added to aid ^1^H-NMR assignments).

The synthesis and characterization of G0 dendrimer conjugates were previously reported [10,11] in a study in which diethylene glycol was used as a linker between naproxen, a poorly water-soluble drug, and G3 PAMAM dendrimer (Figure 1). Naproxen-diethylene glycol (NAP-deg) was attached to the primary amines of G3 dendrimer using 4-nitrophenyl chloroformate activation. NAP-deg was initially reacted with 4-nitrophenyl chloroformate to obtain NAP-deg-(4-nitrophenyl carbonate) which was purified and characterized by ^1^H-NMR and ^13^C-NMR spectroscopy [10]. G3 dendrimer and NAP-deg-(4-nitrophenyl carbonate) were reacted in appropriate molar ratios to form a carbamate bond between the diethylene glycol linker and the G3 dendrimer. Lauroyl alcohol was attached to a primary amine of the G3 dendrimer-naproxen conjugates through a carbamate bond using 4-nitrophenyl chloroformate activation (Figure 2a).

G3-(deg-NAP)x and LyG3-(deg-NAP)x were purified by size exclusion chromatography, solubilization in water (NAP-deg and lauroyl alcohol are poorly soluble in water) and dialysis (MW cutoff = 3500 Da) for 48 h.

**Figure 3:**
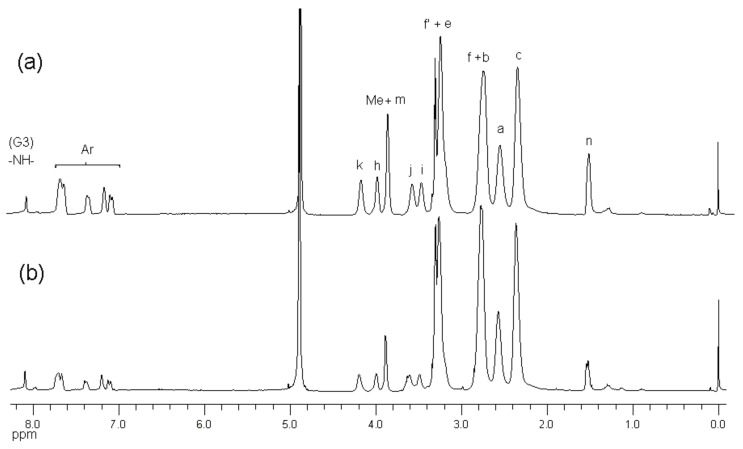
^1^H-NMR (*d*_4_-MeOD) spectra of (**a**) G3-(deg-NAP)10 and (**b**) G3-(deg-NAP)5.

**Figure 4:**
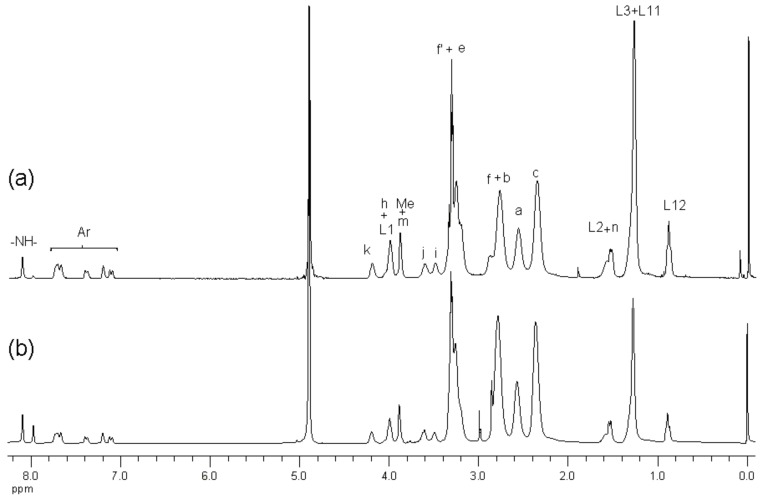
^1^H-NMR (*d*_4_-MeOD) spectra of (**a**) L12G3-(deg-NAP)5 and (**b**) L6G3-(deg-NAP)5.

**Figure 5:**
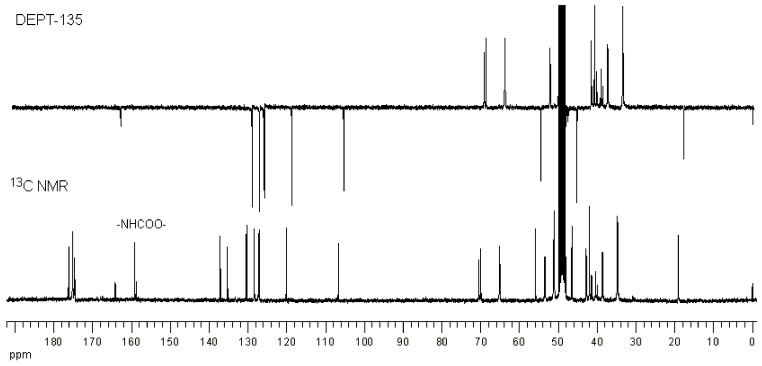
^13^C-NMR and DEPT-135 (*d*_4_-MeOD) spectra of G3-(deg-NAP)10.

^1^H-NMR spectroscopy of the conjugates showed the presence of NAP-deg and/or lauroyl chain and G3 PAMAM dendrimer in the product (Figure 2b, Figure 3 and Figure 4). The formation of a carbamate bond between the diethylene glycol linker and/or lauroyl chain and the amine surface of G3 dendrimer was confirmed by the appearance of a peak(s) at 159.2 ppm on the ^13^C-NMR spectrum, and were assigned with the aid of DEPT-135 (Figure 5). No traces of the free drug, drug linker or lauroyl chain were detected using RP-HPLC of the conjugates (data not shown), which confirmed that naproxen or/and lauroyl chain is covalently (rather than electrostatically) attached to the dendrimer. The drug and lauroyl chain average payloads in the conjugates were estimated using the relative intensities of the peaks in the ^1^H-NMR spectrum originating from attached ligands compared to those of dendrimer (Table 1). It is worth noting that size exclusion was used to produce conjugates with the narrowest possible polydispersity in terms of size and molecular weight.

### 2.2. Determination of Partition Coefficient and Solubility

Solubility is one of the physicochemical parameters that influence drug permeability, and therefore its pharmacokinetic profile and therapeutic activity. In order to increase the solubility of lipophilic naproxen, it was attached to the surface groups of highly water soluble PAMAM dendrimer through a degradable linkage [10]. Since determination of solubility would require the synthesis of large amounts of conjugate, a maximum of 50 mg conjugate per mL was used to evaluate the enhanced solubility of NAP after conjugation at pH 1.2 (i.e., naproxen (pKa 4.2) is not ionised at this pH). The apparent partition coefficients (K_app(o/w)_) of naproxen and conjugates between 1-octanol and phosphate buffer (pH 7.4) were determined as an indicator reflecting the change in the lipophilicity of naproxen after attaching to G0 or G3 PAMAM dendrimers. The conjugation of naproxen to both G0 and G3 dendrimers resulted in a significant decrease in the lipophilicity of naproxen as indicated by the apparent partition coefficients of dendrimer naproxen prodrugs (Table 2). For G0 conjugates, the surface modified conjugate L-G0-deg-NAP showed higher lipophilicity compared to the unmodified surface conjugates G0-deg-NAP (but lipophilicity is still significantly (*p* < 0.05) lower than that of naproxen) as a result of the presence of lipophilic chains (lauroyl chains) on the surface of dendrimer (Table 2). In the case of G3 conjugates, surface modified conjugates (i.e., those having attached lauroyl chains) showed no significant difference (*p* > 0.05) in lipophilicity compared to the unmodified surface conjugates. Apart from Naproxen (pKa 4.2) which is ionised at pH 7.4, there was no significant difference in log K_app(o/w)_ values of the conjugates at both pH 7.4 and pH 1.2.

Correspondingly, the covalent conjugation of naproxen to both generations (G0 and G3) resulted in significant enhancements (*p* < 0.05) in its solubility. The highest solubility of conjugated naproxen is shown for the conjugates that are not surface modified (*p* < 0.05, Table 3). However, attaching more than five naproxen molecules and 12 lauroyl chains to G3 dendrimers resulted in products that were insoluble in water (data not shown). 

### 2.3. The Effect of PAMAM Dendrimer Prodrugs on Caco-2 Cell Viability

**Figure 6:**
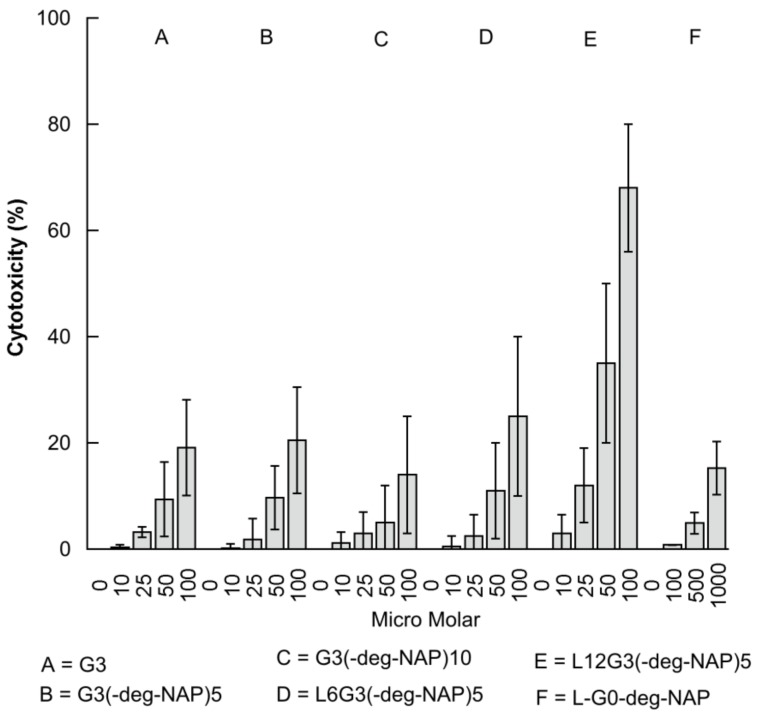
The effect of G3 PAMAM dendrimer, G3-naproxen conjugates (LyG3-(deg-NAP)x), and L-G0-deg-NAP on the viability of Caco-2 cells (LDH assay) (mean ± S.D., *n* = 4).

The influence of PAMAM dendrimer prodrugs on the viability of Caco-2 cells is reported using the LDH assay. LDH, a cytosolic enzyme, has been found to leak out of cells upon damage to cell membranes [12]. G3 PAMAM dendrimer and conjugates showed a concentration-dependent toxicity (Figure 6 and Table 4) with approximately 20% cytotoxicity at concentrations of 100 µM after 180 min incubation. Attaching 5 molecules of naproxen + linker to the surface of G3 PAMAM dendrimer had no significant impact on the cytotoxicity of the resulting prodrug (G3-(deg-NAP)5). However, a significant decrease in cytotoxicity was found after attaching 10 molecules of naproxen + linker to give G3-(deg-NAP)10. The reduced cytotoxicity might result from reduction and shielding of the cationic charges on the dendrimer surface. Similar findings have been previously reported [13,14,15].

As shown in Table 4 (IC_50_ results) and Figure 6, the presence of 6 lauroyl chains increased (*p* < 0.05) conjugate cytotoxicity (L6G3-(deg-NAP)5) and an even more pronounced increase (*p* < 0.05) in cytotoxicity was found when 12 lauroyl chains were attached (L12G3-(deg-NAP)5), which was excluded from transport studies due to its high cytotoxicity. These results may be explained by the intrinsic cytotoxicity of the lauroyl chain [11,13,14,16]. In contrast, previous studies from our group reported that the cytotoxicity of G3 PAMAM can be decreased by modifying it’s surface with 6 lauroyl chains, but was significantly increased when the number of attached chains was increased to 9. The reason for these differences may be that in the earlier work, a 3-(4,5-dimethylthiazole-2-yl)-2,5-diphenyl tetrazolium bromide (MTT) assay was used rather than the LDH assay. A recent study by our group has found that the MTT assay can yield anomalous data in the assessment of high generation dendrimer cytotoxicity in Caco-2 cells (unpublished data). A comparison between the IC_50_ of LG0-deg-NAP and that of G3 PAMAM dendrimer conjugates shows that the G0 prodrug is significantly less toxic (*p* < 0.05) than G3 and its conjugates at the same concentrations (Table 4 and Figure 6). This is in agreement with previous observations which showed that the cytotoxicity of PAMAM dendrimers was due to concentration, generation, and charge dependency [13,17].

### 2.4. Transport of Naproxen, G0 PAMAM Dendrimer and Conjugates across Caco-2 Monolayers

**Figure 7:**
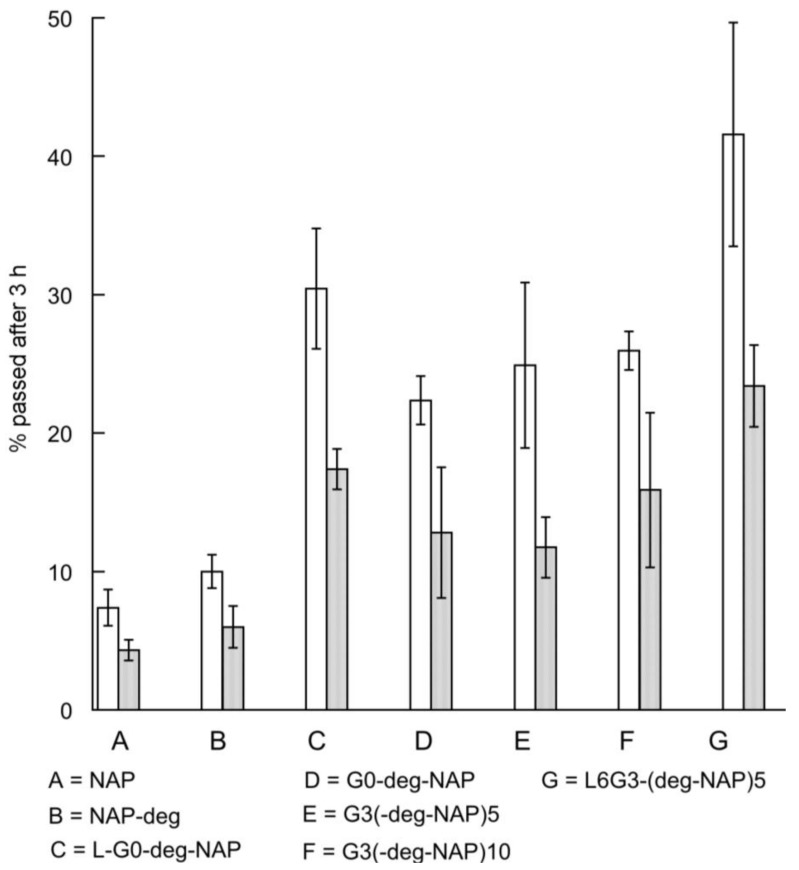
The A-B (□) and B-A (■) transport of naproxen across Caco-2 cell monolayers for naproxen and conjugates (each equivalent to 100 µM naproxen) after 3 h of incubation (mean ± S.D., *n* = 4).

The transport of naproxen, G3 conjugates and L-G0-deg-NAP across Caco-2 monolayers was investigated in both A→B and B→A directions at non-toxic concentrations (as determined by the LDH assay). The concentration of conjugates used in the study were all equivalent to 100 µM naproxen (Table 1). Approximately 8% of the conjugate was hydrolyzed during permeation through the cells to release naproxen, which was detected in the receiver compartment. Therefore, in order to compare transport data for the range of conjugates, permeability was expressed as the percentage of equivalent naproxen transported across Caco-2 monolayers after 180 min (Figure 7) rather than apparent permeability coefficient. 

The transport of G3-(deg-NAP)5 and G3-(deg-NAP)10 across Caco-2 monolayers, especially in the A→B direction, was similar and both were significantly higher (*p* < 0.05) than that of naproxen itself. No significant difference (*p* > 0.05) can be observed between the transport of G3 conjugates (G3-(deg-NAP)5 and G3-(deg-NAP)10) and G0-deg-NAP. These results suggest that the PAMAM dendrimer functions as a drug carrier that is able to cross cellular barriers and enhance the permeability of the drug regardless of the generation. Moreover, these findings are in agreement with previous observations that low generation PAMAM dendrimers (G0 and G1) exhibit significantly less cytotoxicity and higher permeability than higher generations (G2, G3 and G4) [17]. 

A comparison between the transport of naproxen-diethylene glycol (NAP-deg) and naproxen confirms that the enhanced permeability of the conjugate arises from the attachment of the drug to the PAMAM dendrimer. The high permeability of PAMAM dendrimer conjugates may be explained by the presence of positively charged amine surface groups. Knipp et al. [18] reported that positively charged molecules permeate at a higher rate across Caco-2 monolayers compared to neutral or anionic molecules because of the favourable electrostatic interaction with the negatively charged epithelial surfaces. Similarly, Jevprasesphant et al. [13] have found that the permeability of cationic dendrimers (G2, G3, and G4) was higher than that of anionic dendrimers (G2.5 and G3.5).

To enhance the permeability of PAMAM dendrimer conjugates, lauroyl alcohol, a permeability enhancer [19], was used as a surface modifier of G0 and G3 PAMAM dendrimers. As expected, the resulting surface modified G0 and G3 PAMAM dendrimers (L-G0-deg-NAP and L6G3-(deg-NAP)5) showed further enhancement (*p* < 0.05) in the transport of naproxen across epithelial cells (Figure 7). L6G3-(deg-NAP)5 showed the highest transport (*p* < 0.05) in both directions across Caco-2 cell monolayers of all the PAMAM dendrimer conjugates evaluated. However, L-G0-deg-NAP showed an appreciable enhancement of naproxen transport (Figure 7) but is much less cytotoxic (*p* < 0.05) compared to L6G3-(deg-NAP)5 (Table 4). In general, transepithelial transport of dendrimer conjugates has been found to be due to both paracellular and transcellular pathways [16].

## 3. Materials and Methods

### 3.1. Materials

Third generation PAMAM dendrimers (G3) with ethylenediamine cores were purchased from Dendritech Inc. (Midland, MI, USA). Trifluoroacetic acid (TFA), triethylamine (TEA), (S)-(+)-6-methoxy-α-methyl-2-naphthaleneacetic acid (naproxen), 4-nitrophenyl chloroformate, 1-dodecanol (lauroyl alcohol), ethylenediamine tetraacetic acid (EDTA), lactate dehydrogenase assay kits, trypan blue, Sephadex 25, and Sephadex LH-20 were purchased from Sigma-Aldrich Co. Ltd. (Poole, Dorset, UK). Cell culture materials were from Gibco BRL Life Technologies (Paisley, Scotland). Polycarbonate cell culture inserts (Transwell^®^ 12 mm diameter) and cluster plates (96 well) were purchased from Corning Costar UK (High Wycombe, Bucks, UK). Naproxen-diethylene glycol (NAP-deg), NAP-deg-(4-nitrophenyl carbonate), lauroyl (4-nitrophenyl carbonate), G0-diethylene glycol-naproxen (G0-deg-NAP) and lauroyl-G0-diethylene glycol-naproxen (L-G0-deg-NAP) were synthesized and characterized as previously described [10,11]. G3-naproxen conjugates were characterized using ^13^C and ^1^H-NMR spectroscopy (300 MHz, Bruker Avance 300, Bruker, Coventry, UK). ^13^C-NMR spectra were assigned with the aid of DEPT-135. HPLC analyses were carried out using a Hewlett-Packard Series II 1090 (Hewlett-Packard, Waldbronn, Germany) instrument equipped with a Luna 5 µm, C18 column (250 × 4.6 mm, Phenomenex, Cheshire, UK). The solvent system for characterization of the conjugates was methanol:ACN:H_3_PO_4_ (0.05% *w*/*v*) (10:32:58) for 2 min then (48:32:20) for the remaining elution time. The flow rate was 1.2 mL/min and UV detection was at λ = 230 nm. HPLC solvent systems for the apparent partition coefficients and transport studies: methanol:aq H_3_PO_4_ (0.05% *w*/*v*) (80:20) using phenanthrene as an internal standard. The flow rate was 1.2 mL/min and UV detection was at λ = 230 nm.

### 3.2. Synthesis of G3-(Diethylene glycol-naproxen)x (G3-(deg-NAPx))

In order to yield the target ratio NAP:G3 in anhydrous dimethylformamide (DMF) (1 mL), 10% excess of the required amount of NAP-deg-(4-nitrophenyl carbonate) was added dropwise over 2 hours (h) to a stirred solution of G3 dendrimer in DMF (2 mL). The reaction mixture was stirred for 5 days. DMF was evaporated under vacuum. The residue was dissolved in 3 mL water and filtered. The filtrate was purified by size exclusion chromatography, using Sephadex 25, eluting with methanol:water (1:5). G3-(deg-NAP)5 and G3-(deg-NAP)10 were synthesized and characterized by ^1^H-NMR and ^13^C-NMR spectroscopy.

### 3.3. Synthesis of (Lauroyl)y-G3-(diethylene glycol-naproxen)x (LyG3-(deg-NAP)x)

In order to yield the target ratio L:G3 in 1 mL DMF, 10% excess of the required amount of lauroyl (4-nitrophenyl carbonate) was added dropwise over 2 h to a stirred solution of G3-(deg-NAP)5 in DMF (2 mL). The reaction mixture was stirred for 5 days. DMF was evaporated under vacuum and the residue was purified by size exclusion chromatography using Sephadex LH 20 with methanol:water (5:1 *v*/*v*). The resulting product was dissolved in water and filtered. The filtrate was concentrated under vacuum and purified again by size exclusion chromatography using Sephadex 25, eluting with methanol:water (1:5 *v*/*v*). L6G3-(deg-NAP)5, L12G3-(deg-NAP)5, were synthesized and characterized by ^1^H-NMR and ^13^C-NMR spectroscopy (Bruker Avance 300, Bruker, Coventry, UK). 

### 3.4. Determination of Partition Coefficients and Soluiblities

The apparent partition coefficients (K_app(o/w)_) of G0 and G3 dendrimer conjugates between 1-octanol and phosphate buffer (pH 7.4) and pH 1.2 (0.06 M hydrochloric acid buffer) were determined at 37 °C. Before use, the 1-octanol was saturated with phosphate buffer for 24 h by stirring vigorously. A known amount of conjugate in phosphate buffer (pH 7.4, 5 mL) was shaken for 72 h with 1-octanol (5 mL) to achieve equilibrium, and the phases were separated by centrifugation at 10,000 revolution per minute (rpm) for 5 minutes (min). All experiments were performed in triplicate. The concentrations of the compounds in the buffer phase before and after partitioning were determined by HPLC (Hewlett-Packard, Waldbronn, Germany). 

The solubility of G0 and G3 dendrimer conjugates in pH 1.2 (0.06 M hydrochloric acid buffer) were determined at 37 °C. All synthesized amount of conjugate (50 mg) added to the buffer (pH 1.2, 1 mL) was shaken for 72 h to achieve saturation. The unsolubilised conjugate was then filtered (Millipore, Feltham, UK, 0.45 μm). All experiments were performed in triplicate. The concentrations of the compounds in the buffer were determined by HPLC.

### 3.5. Lactate Dehydrogenase Leakage (LDH) Assay

Human intestinal adenocarcinoma cells (Caco-2) (passage 110–120) were seeded at 10,000 cells/well in 96-well plates and maintained at 37 °C in an atmosphere of 5% CO_2_ and 95% relative humidity in Dulbecco’s Modified Eagle’s Medium (DMEM) supplemented with 1% foetal bovine serum, 1% non-essential amino acids, 50 IU/mL penicillin. and 50 mg/mL streptomycin. After 24 h the medium was removed and the cells were washed in phosphate buffered saline and 200 µL Hanks Balanced Salt Solution (HBSS) containing G3 PAMAM dendrimer, conjugates or G0 PAMAM dendrimer at different concentrations (concentrations were calculated using the average molecular weight (Table 1) for each conjugate as a whole macromolecule). Caco-2 cell monolayers were also treated with blank HBSS and 1% Triton X-100 as low and high controls, respectively. After 3 h of incubation at 37 °C, 100 µL/well supernatant was removed carefully and transferred into corresponding wells of an optically clear 96-well flat bottom microplate. LDH leakage in the compartment was quantified using an LDH assay kit. 

The percentage was calculated using the following equation:

Cytotoxicity (%) = ((exp. − low control)/(high control − low control)) × 100

where exp. is the experimental value.

### 3.6. Transport Studies of G3 PAMAM Dendrimer and Conjugates

Caco-2 cells (passage 83–92 for G0 conjugates and 115–122 for G3 conjugates) were seeded onto polycarbonate 12-well Transwell filters (pore size 3.0 µm, at a density of 1.2 × 10^5^ cells/cm^2^. Cells were grown at 37 °C in an atmosphere of 5% CO_2_ and 95% relative humidity in Dulbecco’s Modified Eagle’s medium (DMEM) supplemented with 10% foetal bovine serum, 1% non-essential amino acids, 50 IU/mL penicillin and 50 mg/mL streptomycin. The medium was changed on alternate days for 21–22 days. The integrity of cells was assessed by measuring the transepithelial electrical resistance (TEER) using a voltohmmeter (EVOM, World Precision Instruments, Sarasota, FL, USA) before and after the experiments. Prior to transport experiments, cells were equilibrated with HBSS for 20 min at 37 °C, and the TEER was determined. The TEER value, corrected for the blank filter resistance, was in the range of 800–950 Ω·cm^2^. Only confluent monolayers were used for the transepithelial transport studies. Transport of compounds was determined in both apical-to-basolateral (A→B) and basolateral-to-apical (B→A) directions. The transport medium (TM) was HBSS with 25 mM *N*-2-hydroxyethylpiperazine-*N*′-2-ethanesulfonic acid (HEPES) and was placed in the donor and receiver compartments. Naproxen, G0 or G3 dendrimer conjugates (each equivalent to 100 µM of naproxen, Table 1) were placed in the donor compartment, and cells were incubated in a humidified atmosphere at 37 °C. TEER was measured every 30 min during the experiment, samples (50 µL) were removed from the receiver compartment at time zero, and after 60, 120 and 180 min, and from the donor compartment after 180 min. In order to calculate the percentage of naproxen released from the conjugate during transport studies, an additional sample was taken from the receiver after 180 min, and TFA was added to release all conjugated naproxen. The samples were analyzed by HPLC.

### 3.7. Statistical Analysis

All experiments were performed at least three times using at least three different batches and the results are presented as the mean ± SD. A one-way analysis of variance (ANOVA) statistical test was performed to allow comparison of more than two groups and Student’s *t*-test was conducted to compare two sets of results. The differences were considered to be statistically significant if the *p*-value was ≤ 0.05.

## 4. Conclusions

The synthesis, characterization, cytotoxicity and transport studies of G3 PAMAM dendrimer-naproxen conjugates using diethylene glycol as spacer/linker are reported. Partition coefficient results indicate that G3-naproxen conjugates were significantly more hydrophilic than the parent drug. A comparison was made between the cytotoxicity and transport profiles of G3 dendrimer conjugates and that of G0 dendrimer conjugates. Cytotoxicity studies showed that G0 dendrimer conjugates were only slightly toxic towards Caco-2 monolayers, and their toxicity was significantly lower than that of G3 dendrimer conjugates. Conjugation of naproxen to G0 and G3 PAMAM dendrimer significantly increased its permeability in both directions across Caco-2 monolayer. A more pronounced increase of naproxen transport was observed when lauroyl chains were used as surface modifiers. No significant difference was shown between the transport profiles of G3 and G0 PAMAM conjugates. However, surface modified G3 dendrimer conjugates showed a higher permeability and significantly higher cytotoxicity than that of surface modified G0 dendrimer conjugates. Future investigations may focus more on the impact of conjugation on the density of surface charge for dendrimer conjugates and the aggregation behaviour of these conjugates in aqueous solutions. 

Overall, our results suggest that G0 PAMAM dendrimers demonstrate potential as nanocarriers for the enhancement of oral bioavailability of naproxen as a model for low aqueous solubility drugs.

## Figures and Tables

**Table 1:** Conjugation ratios and concentrations PAMAM dendrimer conjugates used in the transport studies.

	Conjugation Ratio G3:Naproxen ^†^	Conjugation Ratio G3:Lauroyl Chain ^†^	Average wt/mol (g/mol)	Conjugate Concentration (µM)	Equivalent Concentration of Naproxen (µM)
G3	n/a	n/a	6909	n/a	n/a
G3-(deg-NAP)5	1:5.2	n/a	8636	19.2	99.8
G3-(deg-NAP)10	1:10.5	n/a	10,363	9.5	99.8
L6G3-(deg-NAP)5	1:4.8	1:6.4	9916	20.8	99.8
L12G3-(deg-NAP)5	1:5.4	1:11.6	11,196	n/a	n/a
G0	n/a	n/a	517	n/a	n/a
G0-deg-NAP	1:1	n/a	861	100.0	100.0
L-G0-deg-NAP	1:1	1:1	1073	100.0	100.0

^†^ Determined by ^1^H-NMR spectroscopy.

**Table 2:** The log K_app(o/w)_ (pH 7.4 and pH 1.2) values of naproxen and its conjugates at 37 °C.

Compound	pH 7.4	pH 1.2
NAP	0.11 ± 0.01	2.8 ± 0.7
G0-deg-NAP	−1.07 ± 0.09	−1.17 ± 0.1
L-G0-deg-NAP	−0.19 ± 0.07	−0.18 ± 0.05
G3-(deg-NAP)5	−0.36 ± 0.12	−0.35 ± 0.19
G3-(deg-NAP)10	−0.40 ± 0.13	−0.37 ± 0.17
L6G3-(deg-NAP)5	−0.40 ± 0.09	−0.32 ± 0.11
L12G3-(deg-NAP)5	−0.18 ± 0.04	−0.17 ± 0.09

**Table 3:** The solubility (pH 1.2) naproxen and its conjugates at 37 °C.

Compound	Solubility mg/mL	Solubility Eq. con. NAP * (mM)
NAP	0.06	0.26
G0-deg-NAP	>50	>58
L-G0-deg-NAP	35 ± 12	32 ± 11
G3-(deg-NAP)5	>50	>28
G3-(deg-NAP)10	>50	>48.2
L6G3-(deg-NAP)5	29 ± 17	14 ± 8.2
L12G3-(deg-NAP)5	18 ± 12	8 ± 5.7

* Equivalent concentration of naproxen (mM).

**Table 4:** The effect of G3-naproxen conjugates (LyG3-(deg-NAP)x) and L-G0-deg-NAP on the viability of Caco-2 cells as determined by IC_50_ (mean ± S.D., *n* = 4).

Compound	IC_50_ (µM)
G3	247 ± 35
G3-(deg-NAP)5	225 ± 32
G3-(deg-NAP)10	357 ± 57
L6G3-(deg-NAP)5	189 ± 28
L12G3-(deg-NAP)5	76 ± 15
L-G0-deg-NAP	3181 ± 455
